# Biaxial Flexural Strength of High-Translucence Monolithic Ceramics upon Various Thicknesses

**DOI:** 10.1155/2021/4323914

**Published:** 2021-10-06

**Authors:** Niwut Juntavee, Apa Juntavee, Sirintana Phetpanompond

**Affiliations:** ^1^Department of Prosthodontics, Faculty of Dentistry, Khon Kaen University, Khon Kaen 40002, Thailand; ^2^Division of Pediatric Dentistry, Department of Preventive Dentistry, Faculty of Dentistry, Khon Kaen University, Khon Kaen 40002, Thailand; ^3^Division of Biomaterials and Prosthodontics Research, Faculty of Dentistry, Khon Kaen University, Khon Kaen 40002, Thailand

## Abstract

**Introduction:**

High-translucence ceramics have been used increasingly. This study evaluated the biaxial flexural strength of different ceramics as a result of varying thicknesses.

**Materials and Methods:**

Circular discs with varied thickness of 0.4, 0.6, 0.8, 1.0, 1.2, 1.4, 1.6, 1.8, and 2.0 mm were prepared from high-translucence yttria-partially stabilized zirconia (HTY-PSZ); Bruxzir^®^ Anterior (Bc), and zirconia-reinforced lithium silicate (ZLS) including Celtra^®^ DUO (Cc) and VITA Suprinity^®^ (Vc) (*n* = 15 discs/group). Biaxial flexural strength (*σ*) was evaluated utilizing piston-on-three-balls in a testing machine at a speed of 0.5 mm/min. A scanning electron microscope (SEM) was used to determine the microscopic structure. ANOVA and multiple comparisons were analyzed for significant differences (*a* = 0.05).

**Results:**

The mean ± sd value of *σ* (MPa) for thickness 0.4, 0.6, 0.8, 1.0, 1.2, 1.4, 1.6, 1.8, and 2.0 mm was 672.66 ± 107.54, 655.93 ± 93.98, 589.01 ± 63.63, 624.89 ± 87.08, 618.82 ± 83.36, 672.64 ± 84.61, 659.81 ± 122.89, 632.79 ± 92.54, and 657.86 ± 73.17, for Bc; 477.64 ± 88.23, 496.39 ± 86.36, 461.56 ± 57.00, 450.26 ± 86.60, 468.28 ± 83.65, 472.45 ± 53.63, 453.05 ± 72.50, 462.67 ± 47.57, and 535.28 ± 84.33, for Cc; and 500.97 ± 76.36, 506.70 ± 87.76, 557.82 ± 62.78, 543.76 ± 87.29, 507.53 ± 86.09, 502.46 ± 64.75, 557.70 ± 80.91, 527.04 ± 80.78, and 499.88 ± 57.35, for Vc. A significant difference in flexural strength was indicated among groups (*p* < 0.05). Bc was significantly stronger than Cc and Vc (*p* < 0.05). Varying thickness did not have a significant influence on strength (*p* > 0.05). SEM revealed a tight arrangement of crystals for Bc and needle-like crystals diffusing in glass for Vc and Cc.

**Conclusion:**

Flexural strength of ceramics varied among types, but each retained strength equitably with varying thickness. HTY-PSZ was stronger than ZLS, but each was equally strong for thickness in the range of 0.4–2.0 mm.

## 1. Introduction

The evolution of computer-assisted design and computer-assisted manufacturing (CAD-CAM) systems has started to play an important role in the contemporary practice of restorative dentistry as a result of modern technology, instruments, materials, and treatment techniques that are capable of providing a natural esthetic restoration within a short period of treatment. This includes the advancement of the digital approach for chairside CAD-CAM fabricated zirconia ceramic restorations in conjunction with the rapid-sintering process that leads to the fabrication of ceramic restoration becoming a more dehumanized, efficient, and precise procedure [1]. The supreme concern for restorative dentistry is to search for and select ceramic materials that possess superior esthetics and high durability. Among all ceramic materials, 3 mol% (5.2 wt%) yttria-stabilized tetragonal zirconia polycrystal (3Y-TZP), containing 0.25 wt% aluminous oxide (Al_2_O_3_), has been gaining attention due to its exceptional mechanical and biological properties that can produce restoration with appropriate esthetics to replicate natural dentition. Even though 3Y-TZP possesses high strength, it has an inherently brittle property and is extremely opaque because of its inherently birefringent phenomenon from mainly tetragonal (*t*) and monolithic (*m*) phase composition, inducing the light scattering effect at the grain boundaries, pores, and other material inclusions. Therefore, the classical zirconia needs to be veneered with feldspathic glass-ceramic to produce porcelain veneered zirconia (PFZ) to obtain a natural translucent appearance of the tooth. However, long-term failures of PFZ, mainly associated with chipping and delamination of veneering glass-ceramic, have been evidenced [2]. The primitive monolithic translucence zirconia, such as classical ceramic veneered zirconia, was evolved primarily to withstand high loads during a masticatory function at low risk of failure. The traditional translucence monolithic zirconia retained mechanical properties comparable to those of the classical zirconia regarding resistance to fracture, by maintaining the property of phase transformation from t-⟶m-phase and inducing compressive stress to reduce the fracture size, thus enhancing fracture strength, known as the transformation toughening phenomenon [2, 3]. The esthetic translucence actualization of traditional monolithic zirconia is derived from the reduction in the amount of aluminous oxide composition in conjunction with the repositioning of Al_2_O_3_ particles shifting to the grain boundaries and eliminating the number and size of the internal porosities by modifying sintering processes, such as increasing the sintered temperature and sintering time [3]. However, the translucence of traditional monolithic zirconia was rather unpredictable, and therefore, its usage was preferred for restoration in the posterior region. Recently, highly translucence monolithic yttria-partially stabilized zirconia (HTY-PSZ) has been introduced with a higher percentage of yttrium oxide (Y_2_O_3_) content to produce more translucency of yttria-partially stabilized zirconia, such as 4 mol% of yttrium oxide (4Y-PSZ) and 5 mol% of yttrium oxide (5Y-PSZ), which leads to the microscopic composition comprising high amounts of cubic (c-) phase [4, 5]. The amount of c-phase produced significantly improves the translucence of zirconia; nonetheless, its rather diminishing fracture strength and toughness, compared to classical zirconia, is due to the fact that the c-phase rarely exhibited the stress-induced transformation toughening phenomenon [4]. Therefore, the HTY-PSZ materials were indicated for use in a highly esthetic region.

Esthetic dentistry is still witnessing a trend toward monolithic ceramic restorations, together with the evolution of chairside digital scanning cameras and computerized control milling and processing processes for the fabrication of ceramic restorations from sintered or partially sintered blanks. Fundamentally, the tradeoff of ceramics with the highest durability with ceramics with poor translucence becomes obvious, which is driving the need for a new monolithic ceramic material. Consequently, glass-ceramics have been gaining popularity for monolithic ceramic restoration due to their superior translucence property, which is attributed to their composition being primarily glass-producing esthetics similar to natural dentition. The classical glass-ceramics comprise a lithium disilicate (LS_2_) crystalline structure embedded in a Li_2_O-2SiO_2_ glass matrix containing up to 4 wt% zirconium oxide (ZrO_2_) functioning as dispersion strengthening particles. Recently, a new lithium-based glass-ceramic containing a thermodynamic stable lithium silicate (LS) crystal structure, comprising more lithium metasilicate crystalline structures, which included 10 wt% ZrO_2_, evidently as a reinforcement structure, namely, zirconia reinforce lithium silicate (ZLS), was introduced by combining the high esthetic achievement of lithium disilicate glass-ceramic with the strengthening process from zirconia content [6–8], providing better esthetics than classical translucence Y-TZP [9, 10]. ZLS was developed by adding more zirconia to LS_2_ glass-ceramics to achieve better resistance to heavy occlusal force over the original glass-ceramic by subsuming the zirconia contents into the glass-forming matrix as lithium metasilicate and lithium disilicate biphase ceramic structures. Several studies reported that the strength of ZLS is higher than that of some classical glass-ceramics [2, 11–13]. This leads to a competition of using either HTY-PSZ ceramic or ZLS ceramic in clinical practice [12].

Nevertheless, it is still challenging for dentists to treat patients successfully by utilizing these materials. The dentist must determine the appropriate material and design for each type of restoration for each patient. The search for materials that can be made for a thin area such as veneer, inlay, onlay, and even some thin areas of a crown or bridge, such as those at the margin, can still be a major concern. The thickness of restorations may influence the durable ability to withstand the ceramic fracture [14–19]. Previous studies found that the thickness of ceramic strongly influenced the strength of ceramic restoration [13, 20, 21]. The thickness of 0.5–1 mm was recommended for the margin of all ceramic restorations with a slightly rounded internal angle to reduce the stress at the line angle that frequently initiated fracture [22, 23]. A recent study reported that the flexural strength of conventional monolithic zirconia with 0.8–1.3 mm thickness was greater than the human force of mastication [24]. Other studies recommended that ZLS with 1.0–1.5 mm thickness should be used in practice [11, 25]. Conversely, some studies reported that the thickness of the ceramic restoration did not affect the flexural strength [26–29]. The difference in the study designs of previous studies probably led to dissimilar results. This leads to confusion when clinicians are seeking appropriate guidelines for practice in ceramic restoration in terms of ceramic thickness, especially concerning HTY-PSZ and ZLS.

Ceramic in dental treatment is always faced with some areas with sufficient space for ceramic restoration, whereas others manifest with a thin area for the feasible thickness of ceramic restoration. The thin area of ceramic restoration, for example, at the margin, is still the major concern of the clinician in using ceramics in treatment. A few studies reported about the thickness of ceramic restoration or the space required for ceramic restoration that ensures resistance to chipping or fracture before the process of cementation ceramic restoration to the prepared abutment [30]. Most dentists are concerned that their ceramic restoration may break off during the trial process of restoration on the prepared abutment. Moreover, there is a lack of information about the thickness of translucence monolithic ceramic materials, especially HTY-PSZ and ZLS, that adequately assures strength for the trial process before cementation. As such, this study aimed to compare the flexural strength of HTY-PSZ and ZLS concerning the difference in ceramic thickness. The null hypotheses were the difference in the type and thickness of monolithic ceramics would not lead to different flexural strengths.

## 2. Materials and Methods

### 2.1. Zirconia Disc Preparation

**Figure 1:**
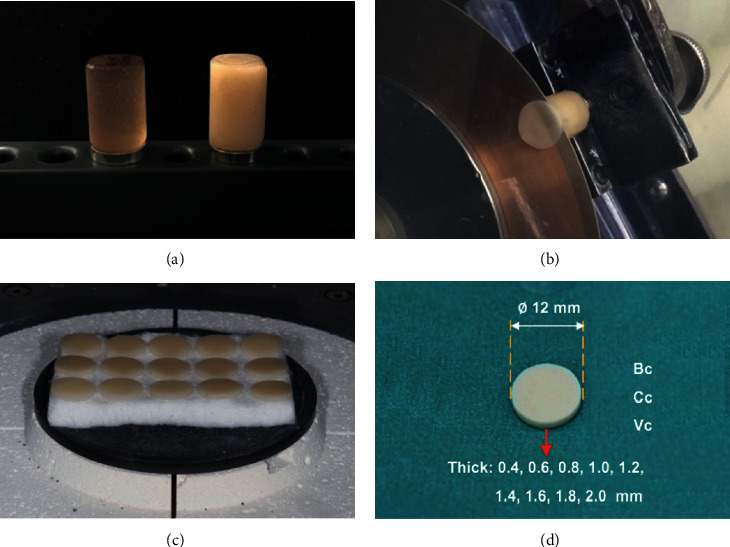
Ceramic was milled in a cylindrical shape (a), sectioned into circular discs (b), and sintered in the furnace (c) to derive for circular discs of 12 mm diameter with a thickness of 0.4, 0.6, 0.8, 1.0, 1.2, 1.4, 1.6, 1.8, and 2.0 mm (d).

The ceramic blanks were milled into a cylindrical shape with 12 mm diameter from monolithic HTY-PSZ using Bruxzir^®^ Anterior (Bc; Glidewell Dental Laboratory, Newport Beach, CA) and from ZLS using Celtra^®^ DUO (Cc; Dentsply, Hanau-Wolfgang, Germany) and VITA Suprinity^®^ (Vc; Vita Zahnfabrik, Bad Säckingen, Germany) and sectioned into a circular disc shape by using a low-speed diamond saw (Isomet^®^ 4000, Buehler, Lake Buff, IL) as illustrated in Figures 1(a) and 1(b). The discs were ground flat on both surfaces by silicon carbide abrasive paper # 360, 600, 800, 1500, and 2,000 and then polished by 1 *µ*m diamond suspension using a polishing machine (Ecomet^®^3, Beuhler) to achieve the desired dimension. All specimens were cleaned in distilled water for 15 minutes using an ultrasonic cleaner (Vitasonic-II, Vita Zahnfabrik) and then allowed to dry at room temperature for 60 minutes. The Bc-specimens were sintered in a furnace (inFire HTC, Sirona, Bensheim, Germany) at a sintering temperature of 1580°C for 150 min holding time with 15°C/min sintering rate. The Cc-specimens were sintered in a porcelain furnace (Programat P-310, Ivoclar Vivadent, Schaan, Liechtenstein) at a sintering temperature of 820°C for 1 min holding time with 60°C/min sintering rate. The Vc-specimens were sintered in a furnace (Programat P-310, Ivoclar Vivadent) at a sintering temperature of 840°C for 8 min holding time with 55°C/min sintering rate as shown in Figure 1(c). The circular disc thickness of 0.4, 0.6, 0.8, 1.0, 1.2, 1.4, 1.6, 1.8, and 2.0 mm within the error of ±0.05 mm (*n* = 15/group) was fabricated as shown in Figure 1(d). The thickness accuracy was checked by using a digital caliper (INSIZE1112-150, Jiangsu, China).

### 2.2. Determination of Biaxial Flexural Strength

**Figure 2:**
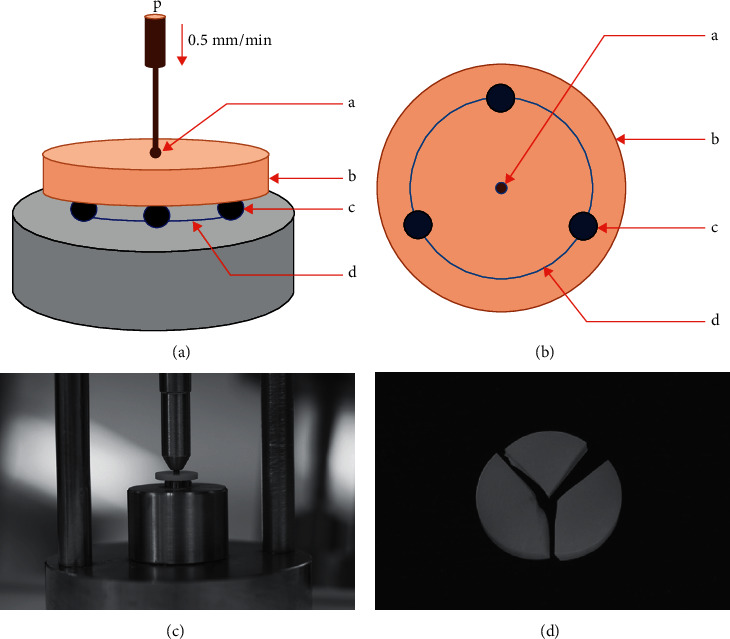
Schematic drawing of the piston-on-three-balls of biaxial flexural strength test (a), (b). Ceramic disc (b) was placed on three balls (c), which were separately arranged in a circular manner at 120° apart from each other (d), and loaded vertically (p) with a round end piston (a) at a speed of 0.5 mm/min until fracture (c). Fracture specimens were further examined microscopically for analysis of fracture (d).

The specimens were tested on the piston-on-three-ball apparatus, as shown in Figure 2. The testing apparatus comprised three spherical steel balls with a diameter of 2.5 mm, which were arranged in a circular shape with a diameter of 11 mm and separately arranged 120° apart from each other, as shown in Figures 2(a) and 2(b). The specimens were placed on three spherical balls and pressed with a piston that had a round end diameter of 1.87 mm. Then, the force was induced from a universal testing machine (LR30/*k*, Lloyd, Leicester, UK) through the piston for loading at the center of the specimen at a rate of crosshead speed of 0.5 mm/min. The load was continuously induced until the ceramic fractured, as shown in Figures 2(c) and 2(d). The load (Newton, N) at failure was recorded and calculated for biaxial flexural strength (*σ*, MPa) using the following equations [29]:

(1) $$\begin{array}{c} \sigma =\frac{\;-0.2378P\left(X-Y\right)}{b^{2}}, \end{array}$$

(2) $$\begin{array}{c} X=\left(1+\upsilon \right)\ln\left[{\left(\frac{r2}{r3}\right)}^{2}\right]+\left[\left(\frac{1-\upsilon }{2}\right){\left(\frac{r2}{r3}\right)}^{2}\right], \end{array}$$

(3) $$\begin{array}{c} Y=\left(1+\upsilon \right)\left(1+\ln\left[{\left(\frac{r1}{r3}\right)}^{2}\right]\right)+\left[\left(1-\upsilon \right){\left(\frac{r1}{r3}\right)}^{2}\right], \end{array}$$

where *P* is a load at fracture (N), *b* is the specimen thickness (mm), *υ* is Poisson's ratio (0.35 for Bc, 0.3 for Cc, and 0.23 for Vc), *r*_1_ is the radius of the support circle (mm.), *r*_2_ is the radius of the loaded area (mm.), and *r*_3_ is the radius of the specimen (mm).

### 2.3. Statistical Analysis

The mean and standard deviation (sd) of biaxial flexural strength at the point of failure (MPa) for each group of monolithic ceramic materials were calculated, compared, and then, further analyzed using two-way ANOVA in conjunction with a post hoc Bonferroni multiple comparisons using statistical software (SPSS version 22, Chicago, IL) to determine significant differences in the flexural strength of monolithic ceramic materials with different thicknesses. The result was considered statistically significant at the 95% confidence interval (CI). A Weibull analysis was used to determine the reliability of flexural strength and to estimate characteristic strength (*σ*_o_) as well as the Weibull modulus of fracture (*m*) using Weibull^++®^ statistics (ReliaSoft, Tucson, AZ) according to the following equation:

(4) $$\begin{array}{c} P_{f}\left(\sigma \right)=1-\exp\;{\left(\frac{-\sigma }{\sigma_{0}}\right)}^{m}, \end{array}$$

where *P*_*f*_(*σ*) is the fracture probability, *σ* is the fracture strength, *σ*_0_ is the characteristic strength, and *m* is the Weibull modulus.

### 2.4. Microscopic Examination

The fracture specimens for Cc and Vc were etched with 5% hydrofluoric acid for 1 min to eliminate any glassy content. The specimens were then labeled and coated with gold at a current of 10 mA and a vacuum of 130 m torr for 3 min and then dried in a desiccator cabinet. Finally, the microstructures of the ceramics were evaluated using a scanning electron microscope (SEM, Hitachi, Osaka, Japan). The crystalline phases of the materials were determined by using an X-ray diffractometer (XRD, PANalytical, Empyrean, Almelo, the Netherlands). The crystal structures were examined at a diffraction angle (2*θ* degree) between 20° and 40° with a step size of 0.02° and an interval time of two seconds by using copper k-alpha radiation. The crystalline phase was identified by match-referencing based on the standards database of XRD powder diffraction. The composition of phases for each material was measured according to the intensity of the scanning peaks using X'Pert Plus software (Philips, Almelo, the Netherlands).

## 3. Results

**Figure 3:**
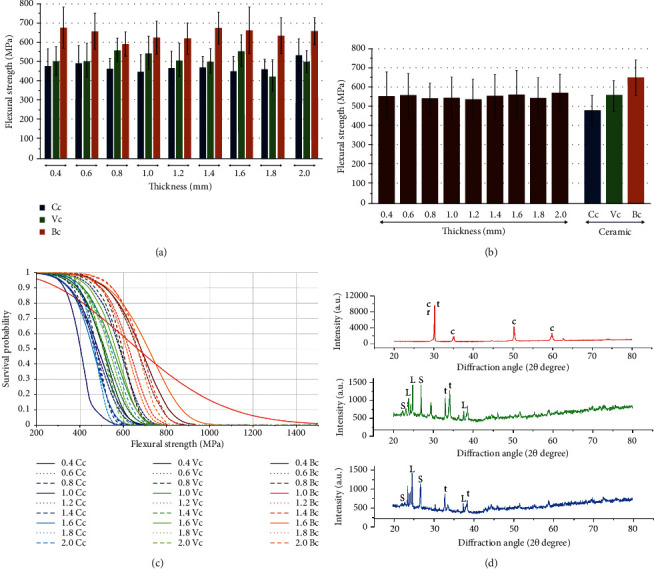
Biaxial flexural strength (a), (b), Weibull survival probability (c), and X-ray diffraction pattern (d) of Celtra^®^ DUO; Cc (a), Vita suprinity^®^; Vc (b), and Bruxzir^®^; Bc (c) at different ceramic thicknesses. t: tetragonal phase, c: cubic phase, r: rhombohedral phase, S: silica oxide, and L: lithium disilicate.

The mean, sd, 95% CI of flexural strength (*σ*, MPa), characteristic strength (*σ*_o_, MPa), and Weibull modulus (*m*) for each group are shown in Table 1 and Figure 3(a). The ceramic material in group Bc demonstrated the highest flexural strength. The ceramic material in group Cc and group Vc demonstrated comparable values of flexural strength. The mean ± sd values of flexural strength for Bc, Cc, and Vc groups were 642.71 ± 92.54, 475.29 ± 76.81, and 522.65 ± 77.98 Mpa, respectively, as shown in Figure 3(b). Two-way ANOVA indicated a statistically significant difference in flexural strength due to the difference in the type of monolithic ceramic materials and their interactions of type of ceramic and ceramic thickness (*p* < 0.05), as shown in Table 2. However, there was no significant difference in flexural strength according to the effect of the ceramic thickness (*p* > 0.05). Post hoc Bonferroni multiple comparisons demonstrated that different monolithic materials showed significant differences in flexural strength (*p* < 0.05), as shown in Table 3 and Figure 3(b). The Bonferroni multiple comparisons of the difference in thickness indicated no significantly different effect on flexural strength (*p* > 0.05), as shown in Table 3 and Figure 3(b). Weibull analysis of the reliability of monolithic ceramic materials upon flexural strength indicated the “m” varied among materials and indicated their relative survival probability of the material upon flexural strength, as shown in Table 1 and Figure 3(c).

**Figure 4:**
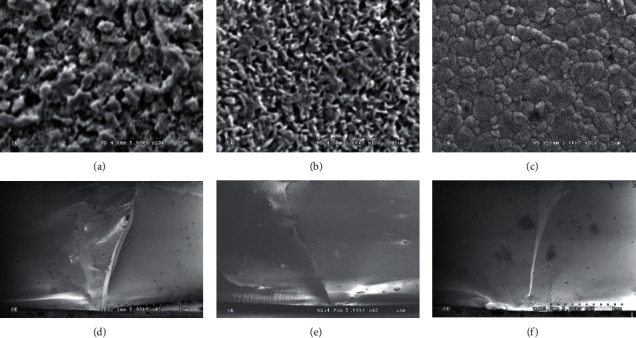
SEM micrographs (X10 K) indicated roundish needle-like crystal contents for Celtra^®^ DUO; Cc (a), Vita suprinity^®^; Vc (b), and multiple crystal structures for Bruxzir^®^; Bc (c). Fractographic micrographs (X45) of Cc (d), Vc (e), and Bc (f) indicated a straight-line crack pattern with narrow flaws.

The XRD analysis of the crystalline contents and phases of the monolithic ceramic materials is shown in Figure 3(d). For the Bc ceramic, the XRD patterns demonstrated a high intensity of t- and c-phases of yttria-partially stabilized zirconia, which were detected at the 2*θ* degree of 30.13°, 34.96°, and 50.19°. The rhombohedral (*r*) or distorted t-phase of zirconia was found at 2*θ* degree of 29.90°. For the Cc and Vc ceramics, the XRD patterns revealed a large amount of crystal structure of silica oxide and lithium disilicate. The crystalline structures of silica oxide were detected at the 2*θ* degree of 21.33° and 27.02° for the Cc and the 2*θ* degree of 21.29° and 26.99° for the Vc. The crystalline structure of lithium disilicate was observed at the 2*θ* degree of 24.88°, 23.82°, 24.39°, and 37.71° for the Cc and at 2*θ* degree of 24.92°, 23.86°, 24.43°, and 37.74° for the Vc. The SEM photomicrographs revealed that, in the Bc ceramic, the crystal structures were arranged tightly within the material structure with an average grain size of approximately 0.8 micrometers (*µ*m). The crystal structure of the Vc was defined as round needle-like crystals of lithium diffusing in the space of glass material with an average grain size of approximately 0.5 *µ*m. The crystal structure of the Vc was found to have an average grain size of approximately 0.5 *µ*m, but the crystal structure of lithium was revealed in the slender appearance and occupied with less amount of porosities than the Cc, as shown in Figures 4(a)–4(c). The similarity in the crack patterns of Bc, Cc, and Vc ceramic specimens was revealed, and the crack propagation tended to demonstrate a straight-line pattern, with sharp and narrow flaws which indicate a brittle nature, as shown in Figures 4(d)–4(f).

## 4. Discussion

This in vitro study found that different types of monolithic ceramic materials demonstrated a significant difference in flexural strength. However, no significant difference in flexural strength regarding the varying thickness of ceramic materials was indicated. Therefore, the null hypothesis was rejected for the type of monolithic ceramic materials but accepted for the variation in thickness. The result of this study clearly shows that HTY-PSZ possessed higher flexural strength than ZLS for both Cc and Vc monolithic ceramic materials. The result of the study is supported by previous studies indicating that the strength of monolithic HTY-PSZ was higher than that of glass-ceramic material [5]. The monolithic HTY-PSZ comprised a primary c-phase with a tightly closed packing of numerous minute grains, leading to stronger fracture resistance than ZLS. Moreover, the monolithic HTY-PSZ still comprised a t-phase that possibly possessed some property in the phase transformation from the t- to the m-phase. The stress-energy from the crack may change the t-crystal to the m-phase, which has a larger volume than the t-phase, so it is more difficult to crack the HTY-PSZ than the ZLS [1, 4]. Both Cc- and Vc-ZLS indicated a high flexural strength value, but when compared to monolithic HTY-PSZ, the result showed slightly lower flexural strength. The Vc-ZLS presented higher flexural strength than the Cc-ZLS, which was possibly related to the difference in their microstructures. The microstructures confirmed by the SEM photomicrograph indicated that the crystal arrangement of Vc was in a thinner needle-like shape and a more diffuse condition than Cc. The fine microstructure and interlocking of LS crystal provided a good mechanical property to resist fracture, thus enhancing the flexural strength of the material [6, 7, 10]. Moreover, Cc comprised more glass volume than Vc, which resulted in enhancing the capability to prevent the crack from growing inside the glass matrix. The SEM photomicrograph indicated sharp and narrow patterns of the crack in each type of monolithic ceramic material, representing the high mechanical property of the brittle structure of these materials [8]. The “m” is also frequently used to describe the characteristics of structural reliability of brittle ceramics. The “m” for each of the tested monolithic ceramics was within the range of 5–15, which was considered acceptable to the average value of most dental ceramics used in dentistry [27]. This indicated the structural reliability of the monolithic ceramics utilized in this study [27, 29].

This study clearly indicated that when the thickness was varied, neither monolithic HTY-PSZ nor ZLS had any significant effect on flexural strength. The result of this study does not correspond with that of some other studies which reported an increase in flexural strength as the thickness of the ceramic increased [1, 13, 21, 24]. This was probably due to the fact that the testing methodology of this study was different from the methodology adopted in the other studies. Moreover, those studies described the strength in terms of the load-bearing capacity of the material, which already integrated the difference in the shape of the specimen into the results. Conversely, this study determined the strength by utilizing the standard method to verify the strength based on the ISO standard number 6782, which outlined the standardized method to clarify strength [29]. Therefore, the findings of this study indicated the validity of the strength of the tested materials. Furthermore, the finding of this study was supported by other studies which found that the biaxial brittle fractures of the different ceramic thicknesses for 8-mol% Y-PSZ were comparable, verifying that the thickness does not affect the flexural strength of ceramics [27, 28]. The biaxial flexural strengths of both HTY-PSZ and ZLS determined by utilizing the piston-on-three-balls test were comparable to those reported in other previous studies, confirming that the reduction of ceramic thickness did not result in a biaxial flexural strength reduction [26, 27]. This study confirmed that the variation in the ceramic thickness of both HTY-PSZ and ZLS did not affect the flexural strength. The study found that the monolithic ceramic materials utilized in this study were capable of withstanding a load of the same level even though the ceramic thickness varied in each group. In addition, the study indicated the reliability of using HTY-PSZ and ZLS ceramics for clinical purposes without any influence from the ceramic thickness. This finding confirms that the restorations fabricated from monolithic HTY-PSZ and ZLS were capable of withstanding the load in clinical practice without any effect from the difference in material thickness. The utilization of either HTY-PSZ or ZLS will be based on the preference of the clinician when selecting a material that is appropriate for the clinical situation.

## 5. Conclusions

The flexural strength of the monolithic HTY-PSZ was stronger than that of the monolithic ZLS. Nevertheless, both monolithic ceramic materials did not indicate any difference in flexural strength as the thickness of the ceramic was varied. Therefore, the strength of monolithic ceramic material depends on the type of ceramic material and not on the thickness of each type of ceramic material. Consequently, dentists should be capable of handling the high-translucence monolithic ceramic restoration with extreme confidence without any concern regarding the effect of the difference in thickness on the strength of the material and selecting an appropriate type of high-translucence ceramic for each specific situation.

## 6. Clinical Significance

This study confirmed that the dentists are capable of using high translucence monolithic ceramics with extreme confidence without any concern regarding the effect of the difference in thickness on the strength of the material and selecting an appropriate type of high-translucence ceramic for each specific situation.

## Figures and Tables

**Table 1:** Mean, standard deviation (sd), 95% confidential interval (CI), characteristic strength (*σ*_o_), and Weibull modulus (*m*) of the flexural strength (MPa) of Celtra^®^ DUO (Cc); Vita suprinity^®^ (Vc); and Bruxzir^®^ (Bc) ceramic at different thicknesses.

Thickness (mm)	Ceramic	Flexural strength	95% CI		*σ*_0_	m
		Mean ± sd	UB	LB		
0.4	Cc	477.64 ± 88.23	428.78	526.51	515.03	5.89
	Vc	500.97 ± 76.36	458.68	543.25	534.09	7.18
	Bc	672.66 ± 107.54	613.11	732.22	719.05	6.83

0.6	Cc	496.39 ± 86.36	448.57	544.22	533.45	6.16
	Vc	506.70 ± 87.76	458.10	555.29	544.21	6.21
	Bc	655.93 ± 93.98	603.88	707.97	696.97	7.65

0.8	Cc	461.56 ± 57.00	429.99	493.13	489.57	7.77
	Vc	557.82 ± 62.78	523.06	592.59	587.53	9.20
	Bc	589.01 ± 63.63	553.78	624.25	617.01	10.33

1.0	Cc	450.26 ± 86.60	402.30	498.21	486.79	5.72
	Vc	543.76 ± 87.29	495.42	592.10	582.37	6.64
	Bc	624.89 ± 87.08	576.67	673.11	777.74	7.75

1.2	Cc	468.28 ± 83.65	421.96	514.61	503.99	5.99
	Vc	507.53 ± 86.09	459.86	555.21	544.72	6.35
	Bc	618.82 ± 83.36	572.66	664.99	654.55	8.27

1.4	Cc	472.45 ± 53.63	442.75	502.15	495.91	9.86
	Vc	502.46 ± 64.75	466.60	538.31	530.30	8.69
	Bc	672.64 ± 84.61	625.78	719.49	709.98	8.70

1.6	Cc	453.05 ± 72.50	412.90	493.19	484.50	6.80
	Vc	557.70 ± 80.91	512.89	602.51	592.33	7.64
	Bc	659.81 ± 122.89	591.76	727.86	715.51	5.38

1.8	Cc	462.67 ± 47.57	436.33	489.02	484.05	10.67
	Vc	527.04 ± 80.78	482.30	571.77	560.95	7.31
	Bc	632.79 ± 92.54	581.54	684.03	640.84	8.59

2.0	Cc	535.28 ± 84.33	488.58	581.98	571.47	6.98
	Vc	499.88 ± 57.35	468.12	531.63	524.97	9.75
	Bc	657.86 ± 73.17	617.34	698.38	690.46	9.88

LB: lower bound, UB: upper bound.

**Table 2:** An analysis of variance (ANOVA) of the flexural strength of Celtra^®^ DUO (Cc); Vita suprinity^®^ (Vc); and Bruxzir^®^ (Bc) ceramics of different thicknesses.

Source	SS	df	MS	F	*p*
Corrected model	2262200.16	26	87007.70	13.15	0.001
Intercept	121127829.95	1	121127829.95	18300.32	0.001
Ceramic	210708.63	16	13169.29	1.99	0.013
Thickness	40470.35	8	5058.79	0.76	0.635
Ceramic *∗* thickness	2011021.18	2	1005510.59	151.92	0.001
Error	2501940.25	378	6618.88		
Total	125891970.36	405			
Corrected total	4764140.41	404			

SS: sum of squares, df: degree of freedom, MS: mean square, F: F-ratio, *p*: *p* value.

**Table 3:** Post hoc Bonferroni multiple comparisons of the flexural strength of Celtra^®^ DUO (Cc); Vita suprinity^®^ (Vc); and Bruxzir^®^ (Bc) ceramics at different thicknesses.

Post hoc multiple comparisons of flexural strength among groups of monolithic ceramic materials									

Group	Bc			Cc			Vc		
Bc	1.000			0.001			0.001		
Cc				1.000			0.001		
Vc							1.000		

Post hoc multiple comparisons of flexural strength among groups of ceramic thickness									

Group	0.4	0.6	0.8	1.0	1.2	1.4	1.6	1.8	2.0
0.4	1.000	1.000	1.000	1.000	1.000	1.000	1.000	1.000	1.000
0.6		1.000	1.000	1.000	1.000	1.000	1.000	1.000	1.000
0.8			1.000	1.000	1.000	1.000	1.000	1.000	1.000
1.0				1.000	1.000	1.000	1.000	1.000	1.000
1.2					1.000	1.000	1.000	1.000	1.000
1.4						1.000	1.000	1.000	1.000
1.6							1.000	1.000	1.000
1.8								1.000	1.000
2.0									1.000

## Data Availability

The data used to support the findings of this study are included in the article.
